# Long-term effectiveness of growth hormone therapy in children born small for gestational age: An analysis of LG growth study data

**DOI:** 10.1371/journal.pone.0266329

**Published:** 2022-04-26

**Authors:** Hae Sang Lee, Change Dae Kum, Jung Gi Rho, Jin Soon Hwang

**Affiliations:** Department of Pediatrics, Ajou University School of Medicine, Ajou University Hospital, Suwon, Korea; PLOS, UNITED KINGDOM

## Abstract

**Purpose:**

Growth hormone (GH) treatment has been used to improve growth in short children who were born small for gestational age (SGA). The aim of this study was to investigate the long-term efficacy of GH treatment in these children.

**Methods:**

Data from a multicenter observational clinical trial (ClinicalTrials.gov NCT01604395, LG growth study) were analyzed for growth outcome and prediction model in response to GH treatment. One hundred fifty-two children born SGA were included.

**Results:**

The mean age of patients born SGA was 7.13 ± 2.59 years. Height standard deviation score (SDS) in patients born SGA increased from -2.55 ± 0.49 before starting treatment to -1.13 ± 0.76 after 3 years of GH treatment. Of the 152 patients with SGA, 48 who remained prepubertal during treatment used model development. The equation describing the predicted height velocity during 1st year of GH treatment is as follows: the predictive height velocity (cm) = 10.95 + [1.12 x Height SDS at initial treatment (score)] + [0.03 x GH dose (ug/kg/day)] + [0.30 x TH SDS at initial treatment (score)] + [0.05 x age (year)] + [0.15 x Weight SDS at initial treatment (score)] ± 1.51 cm.

**Conclusions:**

GH treatment improved growth outcome in short children born SGA. We also developed a prediction model that is potentially useful in determining the optimal growth outcome for each child born SGA.

**Trial registration:**

ClinicalTrials.gov Identifier: NCT01604395.

## Introduction

Small for gestational age (SGA) is a clinical entity defined as newborn infants whose weight and/or length is below the normal for their gestational age and sex [1]. Being born SGA is associated with increased risk of insulin resistance, type 2 diabetes mellitus, lower intelligence, cardiovascular disease, neurodevelopmental impairments, and adult short stature, compared with individuals born appropriate for gestational age [2,3]. Ninety percent of children born SGA eventually show catch-up growth regardless of predisposing factors during the first 2 years of life. However, approximately 10% of children fail to demonstrate catch-up growth, and they remain small throughout childhood and adolescence [4–6].

Several recent studies have shown that growth hormone (GH) treatment is effective to improve adult height in children born SGA without catch-up growth [7]. This treatment was approved by the US Food and Drug Administration in 2001 and by the European Medicines Agency. Since 2014, GH treatment has been covered by medical insurance for short children born SGA older than 4 years of age in Korea. Growth hormone treatment has become much more frequent for SGA children with short stature after the approval of medical insurance in Korea. Although many studies have found that GH treatment is an effective treatment for individuals with SGA who do not experience catch-up growth, there has been no large cohort study of the effectiveness of GH in Korean children born SGA [8,9].

Therefore, the aim of this study was to evaluate the long-term effectiveness of GH treatment in short children born SGA and developed a model to predict individual responsiveness to GH treatment.

## Methods

### Patients

**Fig 1:**
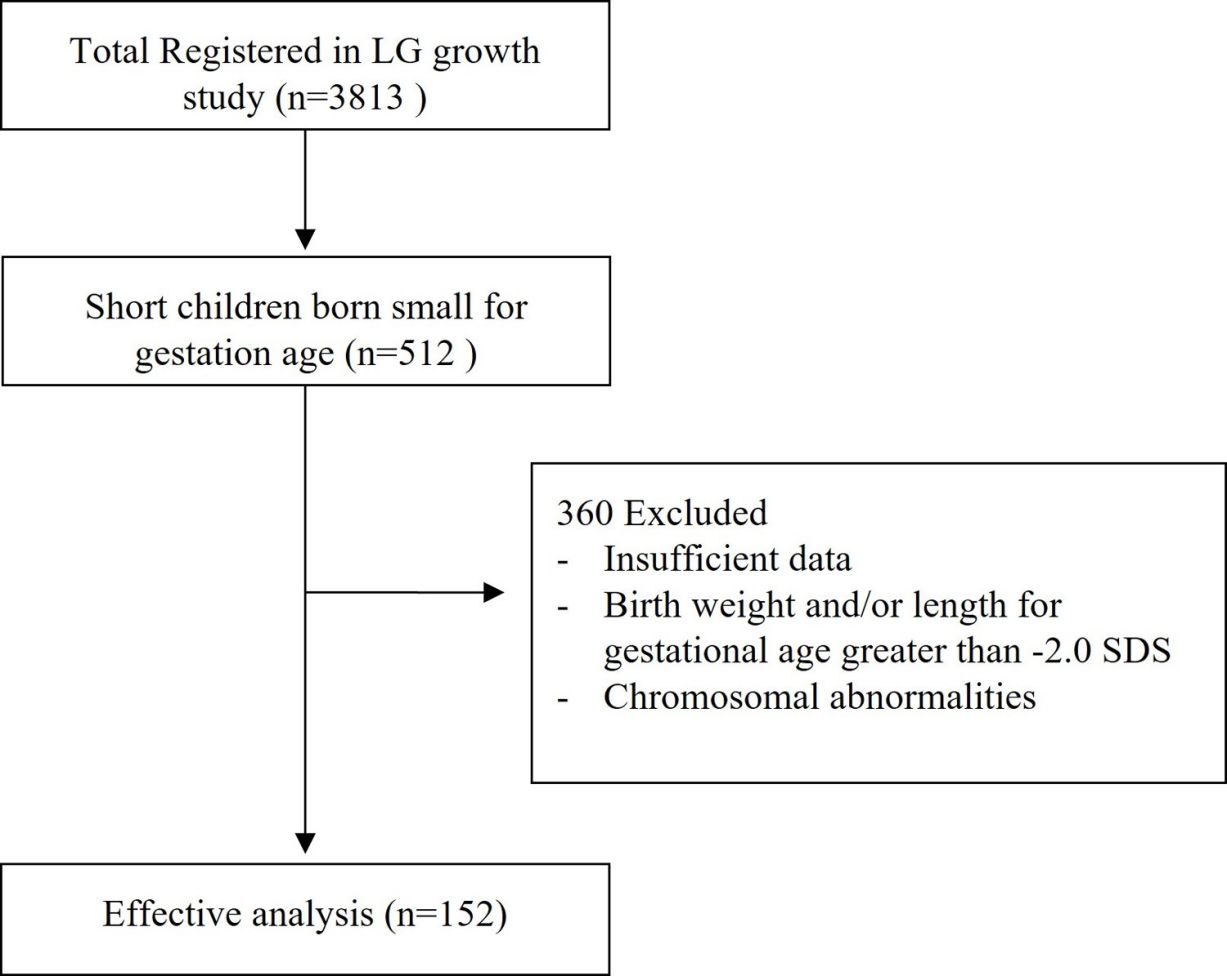
Flow chart of study process.

Patients were screened from the LG growth study (LGS), which is a multi-center, observational study, to analyze the long-term effectiveness and safety of GH (Eutropin inj., Eutropin AQ inj., Eutropin Pen inj. And Eutropin Plus inj.; LG Chem, Ltd., Korea) treatment in patients with GHD, SGA, idiopathic short stature, Turner syndrome, and chronic renal failure [10]. The LGS registry was initiated on 9 November 2011 and the authors accessed data in May 2020. A total of 512 patients with SGA who were registered in LGS between 2011 and 2019 were included in this study. Diagnosis was made according to the LGS etiology classification, as defined by birth weight and/or length for gestational age below -2.0 standard deviation score (SDS) [10,11]. Of the 512 patients, we excluded chromosomal abnormalities and insufficient auxological data (Fig 1). Finally, 152 patients with a height of less than -2.0 SDS after 4 years of age were included.

### Study design

GH was administered subcutaneously at a dose of 33–66 ug/kg/d (initial mean dose: 41.4 ± 1.1 ug/kg/d) for 6 days per week in patients with SGA. GH dose was adjusted based on weight at visits. Patients’ height, weight, bone age, gestational age, birth weight, insulin-like growth factor (IGF)-1, IGF-binding protein-3 (IGFBP-3), pubertal status, serum glucose, and thyroid function were collected from medical records at the time of evaluation and every 6 months. If serum blood glucose was abnormal, oral glucose tolerance test or HbA1c were performed. IGF-1 and IGFBP-3 SDS were calculated using Korean normal IGF-1 and IGFBP-3 levels for age and sex [12]. All laboratory analyses were performed according to local standard procedures of each site (total 73 sites). Target height (TH) was calculated by adding 6.5 cm in boys or subtracting 6.5 cm in girls from mid-parental height. Body mass index (BMI) was calculated using height and weight. To calculate SDS of height, weight, and BMI, we used LMS parameters (Lambda for the skew, Mu for the median, and Sigma) in the 2017 Korean National Growth Charts [13]. Bone age was estimated using the Greulich-Pyle method [14]. Pubertal maturation was determined following the Tanner and Marshall criteria [15]. Prepubertal status was defined as absence of breast development in girls and testicular volume < 4 mL in boys.

### Development of the prediction model

To develop the prediction model, we selected the subjects who were treated with GH for at least 1 year and remained prepubertal during treatment (n = 48). Stepwise multivariable regression analysis was performed using the following independent variables: birth weight, gestational age, age at initiation of treatment, height SDS at start, BMI SDS, TH, GH dose, IGF-1 SDS, and IGFBP-3 SDS. To avoid duplication, we excluded variables with high correlation with suspected co-linearity when variance inflation factor was over 10. For internal validation, the difference between observed and predicted height velocities was expressed in terms of the studentized residuals, as previously published [16]. The studentized residual was calculated as observed height velocity minus the predicted height velocity for each observation and divided by its standard error.

### Ethics approval and consent to participate

Clinical and genetic studies were approved by the Institutional Review Board of the Ajou University Hospital (AJIRB-MED-OBS-20-469). All procedures performed in studies involving human participants were in accordance with the ethical standards of the institutional research committee and with the 1964 Helsinki Declaration. All participants provided written informed consent prior to study participation. Our datasets were obtained from subjects who consented to the use of their individual clinical and genetic data for biomedical research.

### Statistical analysis

Statistical analysis was performed using SAS version 9.4 (SAS Institute, Cary, NC), with P < 0.05 considered statistically significant. Results are reported as mean ± SD unless otherwise noted. To assess differences between groups, we used the independent t test or Mann Whitney U test. Furthermore, the paired t test was performed to evaluate changes in height SDS and growth velocity before and after GH treatment.

## Results

### Subject characteristics

Baseline characteristics of patients with SGA are shown in Table 1. All patients with SGA had a height SDS of less than 2.0 SDS before starting GH treatment. Mean age at initial GH treatment in patients with SGA was 7.13 ± 2.59 years. Of the 152 subjects with SGA, 126 (82.8%) were at a prepubertal stage. Mean gestational age and birth weight were 38.7 ± 1.80 weeks and 2.31 ± 0.42 kg, respectively. Out of 152 subjects with SGA, 55 subjects underwent GH stimulation testing with a combination of at least two of the following: clonidine, dopamine, insulin, and arginine. Ten patients were GH deficient (serum peak GH <10 ng/ml).

**Table 1:** Baseline characteristics in patients born SGA without catch-up growth.

	SGA
	(N = 152)
Age (years)	7.13±2.59
Sex (male, %)	78 (51.4%)
Birth weight	2.31±0.42
Gestational age	38.70±1.80
Height SDS	-2.51 (-2.82, -2.20)
Weight SDS	-2.13 (-2.63, -1.51)
BMI SDS	-0.78 (-1.52, -0.24)
TH SDS	-0.84 (-1.38, -0.52)
Tanner Stage	
Prepubertal	126 (82.8%)
Pubertal	26 (17.2%)
Bone age (years)	6.54±2.81
IGF-1 SDS	-0.55 (-1.25, 0.04)
IGFBP-3 SDS	-0.15 (-1.69, 3.02)
GH dose (μg/kg/day)*	40.9 ± 0.9

Values are represented as mean ± standard deviation and SDS are represented as median (Q1, Q3).

* GH dose refers to the average GH dose over three years.

† The proportion of sex and puberty was compared using the chi square test.

Abbreviation: GH, growth hormone; SGA, small for gestational age; SDS, standard deviation score; TH, target height; IGF-1, insulin growth factor-1.

### Response to GH treatment in patients with SGA

**Fig 2:**
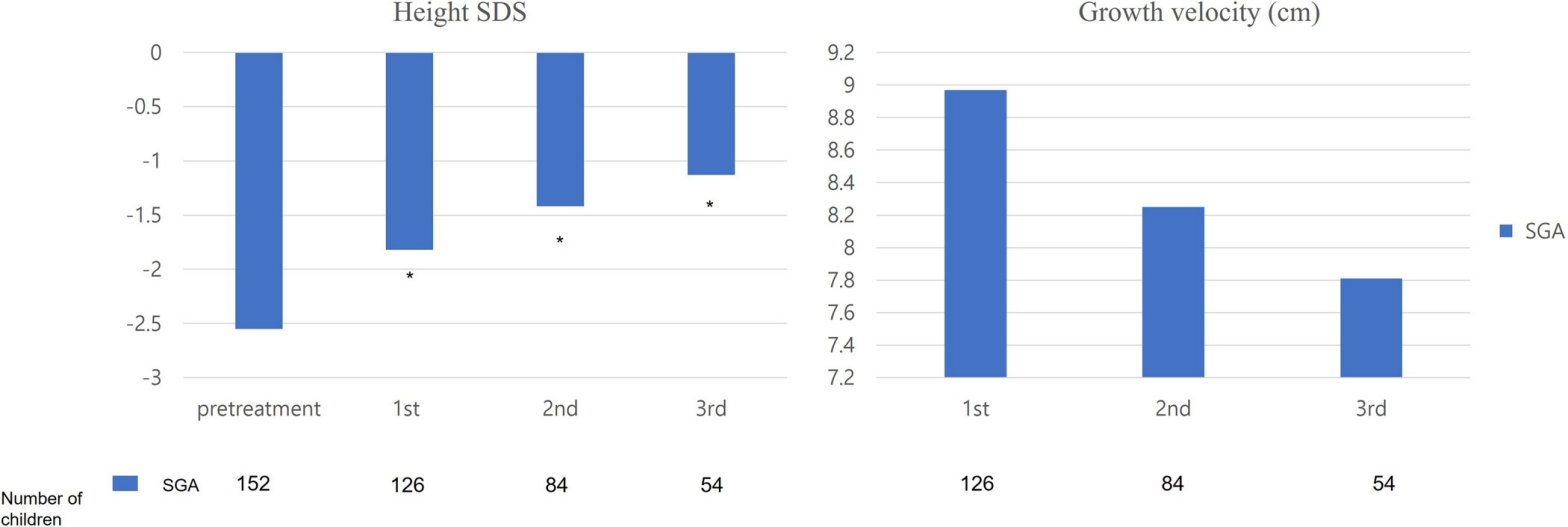
Change in height SDS and growth velocity before and after treatment with GH in children with SGA. * P < 0.001 compared with before growth hormone treatment. Abbreviations: GH, growth hormone; SGA, small for gestational age.

Height SDS in patients with SGA was -1.82 ± 0.65 after first year of treatment, -1.42 ± 0.67 at second year of treatment, and -1.13 ± 0.76 at third year of treatment (Fig 2). Growth velocity in patients with SGA was the highest during the first year of treatment (8.97 ± 1.68 cm), followed by during the second (8.25 ± 1.31 cm) and third years of treatment (7.81 ± 0.80 cm). After 3 years of GH treatment, height SDS in patients with SGA increased from -2.55 ± 0.81 to -1.18 ± 0.89 (p<0.05). Median IGF-1 SDS was 0.72 after first year of treatment, 0.76 at second year of treatment, and 1.29 at third year of treatment. After 3 years of GH treatment, median IGF-1 SDS significantly increased (p<0.05). During study period, no patients with SGA had significant side effects including abnormal glucose metabolism, tumor development, or thyroid hormone abnormalities.

According to pubertal status, we compared the response to GH between pubertal and prepubertal patients in SGA group (Tables 2 and S3). The change of height SDS was not significantly different two groups (0.80 ± 0.33 vs 0.61 ± 018, p = 0.0756). There was no significant different in the height velocity for 1 year between two groups (9.05 ± 1.65 cm vs 8.50 ± 1.80 cm, p = 0.3449). In addition, there was no difference in the response to GH treatment between preterm and term SGA patients (S4 Table). A linear mixed model with pubertal status and time as the fixed effects and growth velocity as the dependent variable was performed. There was no significant interaction between pubertal status and time (S2 Table).

**Table 2:** Change in height SDS and growth velocity before and after treatment with GH according to pubertal status and gestational week in children with SGA (n = 152).

	Prepubertal		Pubertal		
	n	Median (Q1, Q3)	n	Median (Q1, Q3)	P value
Height SDS					
At start	126	-2.51 (-2.82, -2.20)	26	-2.46 (-2.77, -2.37)	0.8072
1^st^ year	48	-1.68 (-2.14, -1.35)	21	-1.94 (2.07, -1.65)	0.3296
Growth velocity (cm)					
1^st^ year	48	9.42 (7.64, 10.24)	21	8.79 (7.76, 9.87)	0.3449
	Preterm (< 37wkees)		Term (≥37 weeks)		
	n	Median (Q1, Q3)	n	Median (Q1, Q3)	P value
Height SDS					
At start	17	-2.55 (-2.82, -2.15)	135	-2.51 (-2.82, -2.21)	0.7880
1^st^ year	15	-1.91 (-2.52, -1.57)	111	-1.70 (-2.07, -1.40)	0.4142
Growth velocity (cm)					
1^st^ year	15	7.80 (6.90, 9.77)	111	9.49 (7.80, 10.03)	0.1198

### Prediction models

Of the 152 patients with SGA, 48 were used for the model development (S1 Table). The parameters found by multiple linear regression analysis, the rank order of importance of the variables as predictors, the overall R^2^, and R^2^ values are presented in Table 3. The equation describing the predicted height velocity during the 1st year of GH treatment is as follows: the predictive height velocity (cm) = 10.95 + [1.12 x Height SDS at initial treatment (score)] + [0.03 x GH dose (ug/kg/day)] + [0.30 x TH SDS at initial treatment (score)] + [0.05 x age (year)] + [0.15 x Weight SDS at initial treatment (score)] ± 1.51 cm. The contribution of the first year response in this model was 21.6% of the total variability. Studentized residual plots showed no values outside -3 and 3 and nonlinearity (S1 Fig).

**Table 3:** Regression equation variables for prediction of the growth response to GH treatment for the first year in short children born SGA (n = 48).

	Parameter Estimate	Standard Error	Partial Variability (R^2^ × 100)	Total Explained Variability (R^2^ × 100)
Intercept	10.95	1.77		21.61
Height SDS at start	1.12	0.54	17.79	
GH Dose (ug/kg/day)	4.88	4.10	2.65	
TH SDS	0.30	0.35	0.64	
Age (year)	0.05	0.10	0.34	
Weight SDS at start	0.15	0.26	0.20	

Abbreviations: GH, growth hormone; SGA, small for gestational age; SDS, standard deviation score; TH, target height.

## Discussion

This analysis of data from the LGS demonstrated that GH treatment improved growth outcomes for children with SGA during the follow-up period. We also developed the prediction model that can be used to predict the first year response to GH treatment in prepubertal children with SGA.

In our study, the mean height gain was 1.42 SDS for 3 years of GH treatment in short children born SGA. Several studies have reported that GH treatment in SGA children without catch-up growth increased height velocity and improves adult height [17–20]. A meta-analysis identified four randomized controlled trials on near adult height in short children with SGA who received GH treatment [21]. From the four trials, the overall mean height gain was 1.5 SDS in GH treated versus 0.25 SDS in untreated SGA children, similar to our results. Recently, Horikawa et al. [17] reported that mean height gain was 1.80 SDS from the start to the end of 5 years of GH treatment. Rapaport et al. [22] also reported that SGA children who received GH treatment for 3 years achieved an increase of 0.8 height SDS, and the mean height gain was not different between children with SGA and those with GHD.

Several factors have been reported to affect GH response in short children with SGA. Age at initiation of GH treatment, height at start of treatment, TH, treatment duration, and GH dose are associated with growth outcome [1]. Consensus guidelines recommend a GH dose range from 35 to 70 ug/kg per day in short children born SGA (6). However, some studies demonstrated an accelerated growth response with higher GH dose, whereas others found similar responses with lower doses. Van Pareren et al. [23] reported a mean height gain from baseline to adult height of 1.8 SDS (a GH dose of 33 μg) and 2.1 SDS (a GH dose of 67 μg) in children with SGA, and adult height SDS was not significantly different between the two GH dosage groups. Tanaka et al. [24] investigated the efficacy of GH treatment with 2 different doses (33 vs 67 μg/kg/day) in children born SGA. They reported that changes in height SDS in children receiving a GH dose of 33 μg/kg/d were lower than those observed in children with a GH dose of 67 μg/kg/d. Lem et al. [25] examined the efficacy of GH treatment with two different doses (1 mg/m^2^/d vs 2 mg/m^2^/d) in patients born SGA. They reported that a GH dose of 2 mg/m^2^/d during puberty results in significantly greater height gain than a GH dose of 1 mg/m^2^/d. In our study, GH was administered at a dose of 40.9 μg/kg/day in children with SGA. Thus, even lower doses of GH, rather than high dose (75 μg/kg/d), improved growth outcome at the beginning of treatment. Further research in a larger cohort is needed to determine the growth outcome according to GH dose.

Our prediction model demonstrated that the most important determinant of first-year growth on GH in children born SGA was the initial height at GH treatment. GH dose, age at initial treatment, TH, and weight SDS were also correlated with growth outcome for the first year of GH treatment. Only few prediction models are available in children with SGA. Ranke et al. [26] developed prediction models for the growth response to GH treatment in short children with SGA using the KIGS database. The prediction factors were age at the initial treatment, weight SDS, TH, and GH dose, similar to that used in this study. In another study, growth prediction models have been used to identify several prediction factors including age at initial treatment, treatment duration, and GH dose [27]. Establishing a predictive model is very important as it can accurately estimate the potential growth of GH treatments and help to optimize GH treatments individually [28]. Subsequent GH dosing may then be changed depending on the desired goals, costs, and observed response to GH treatment. If low responses are predicted, the clinicians may be altered the GH dose in an early stage because of wide range of permitted GH doses.

This study has a few limitations. First, the LGS is an observational study, and variations in data collection may exist because of the large number of participating investigators. Second, we did not evaluate compliance, such as the number of GH injection per week. Third, laboratory parameters including IGF-1 and IGFBP-3 were measured at each site, which may lead to interlaboratory measurement bias. Fourth, sample size used for the prediction model was relatively small. Therefore, the model explained only 21.6% of the variability of the observed growth response. Despite these limitations, our study has strengths in that it is the first multicenter study conducted in Korea. Our findings supported that GH treatment was effective in SGA children without catch-up growth. In addition, the prediction model can help with personalized GH treatment for SGA patients in Korea.

In conclusion, short children born SGA increased height SDS and growth velocity after 3 years of GH treatment. We also developed a prediction model that is potentially useful for determining the optimal growth outcome for each child born SGA. Thereafter, subsequent GH dosing can be altered according to desired objectives and observed response to GH.

## Supporting information

S1 Fig Studentized residuals for prediction model of the change in height velocity. (XLSX)

S1 Table Characteristics of prepubertal children born SGA at the start of GH treatment. (XLSX)

S2 Table Mixed model for height velocity. (XLSX)

S3 Table Baseline characteristics in patients with SGA based on pubertal status. (XLSX)

S4 Table Baseline characteristics in SGA patients based on gestational age. (XLSX)

S1 File Summary of study protocol. (DOC)
