# Supplementary material for: Long-term effectiveness of growth hormone therapy in children born small for gestational age: An analysis of LG growth study data
Source: PLoS One. 2022 Apr 26;17(4):e0266329. doi: 10.1371/journal.pone.0266329 (PMC9041836; doi:10.1371/journal.pone.0266329)
Supplement: S1 File — (DOC) [file pone.0266329.s006.doc]

**Summary of Study Protocol**

| Study title | A multicenter, open, retrospective and prospective observational study to evaluate the long-term safety and effectiveness of growth hormone (Eutropin Inj./EutropinPlus Inj./EutropinAQ Inj./EntropinPen Inj.) with GHD, TS, CRF, SGA, ISS and PWS in children. |
| --- | --- |
| Study purpose | The purpose of this study is to evaluate the long-term safety and effectiveness Eutropin Inj./EutropinPlus Inj./EutropinAQ Inj./EntropinPen Inj) treatment with GHD (Growth Hormone Deficiency), TS (Turner Syndrome),CRF (Chronic Renal Failure), SGA (Small for Gestational Age) and ISS (Idiopathic Short Stature)and PWS(Prader-Willi Syndrome). |
| Study design | An open, multicenter, retrospective and prospective observational study  1) Prospective  Screening visit (obtain informed consent form and confirm inclusion criteria) 🡪 follow-up visit (every 6 months) 🡪 last visit (2 years after epiphyseal closure)  1) Retrospective  Screening visit (obtain informed consent form and confirm inclusion criteria) 🡪 Historical follow-up visit (every 6 months) 🡪 follow-up visit (every 6 months) 🡪 last visit (2 years after epiphyseal closure) |
| Indication | Short stature in children with GHD, TS, CRF, SGA, ISS and PWS |
| Inclusion criteria | **Inclusion criteria**   - 1. Children aged > 2 years   2. Patients were diagnosed with either GHD, TS, CRF, SGA, ISS or PWS   3. Written informed consent form the person, person’s parent or legal guardian   4. Patients who eligible for this study based on the investigator’s judgment |
| Number of patients | 6,000 patients |
| Study duration | Approximately 20 years |
| Study products | Eutropin Inj./EutropinPlus Inj./EutropinAQ Inj./EntropinPen Inj. |
| Administration method | - Eutropin Inj./EutropinAQ Inj./EntropinPen Inj : Once a day, Subcutaneous injection  - EutropinPuls Inj. : Once a weekly, Subcutaneous injection  - Dosage : Individualized treatment for patients |
| Endpoints | Safety  Adverse events(including laboratory test)  Effectiveness  - Difference in target height and final height  - Difference in height velocity between baseline and every year  - Difference in height SDS for CA between baseline and every year |
